# Transcriptomic Response of Nitrosomonas europaea Transitioned from Ammonia- to Oxygen-Limited Steady-State Growth

**DOI:** 10.1128/mSystems.00562-19

**Published:** 2020-01-14

**Authors:** Christopher J. Sedlacek, Andrew T. Giguere, Michael D. Dobie, Brett L. Mellbye, Rebecca V. Ferrell, Dagmar Woebken, Luis A. Sayavedra-Soto, Peter J. Bottomley, Holger Daims, Michael Wagner, Petra Pjevac

**Affiliations:** a University of Vienna, Centre for Microbiology and Environmental Systems Science, Division of Microbial Ecology, Vienna, Austria; b University of Vienna, The Comammox Research Platform, Vienna, Austria; c Department of Crop and Soil Science, Oregon State University, Corvallis, Oregon, USA; d Department of Microbiology, Oregon State University, Corvallis, Oregon, USA; e Department of Biology, Metropolitan State University of Denver, Denver, Colorado, USA; f Department of Botany and Plant Pathology, Oregon State University, Corvallis, Oregon, USA; g Center for Microbial Communities, Department of Chemistry and Bioscience, Aalborg University, Aalborg, Denmark; h Joint Microbiome Facility of the Medical University of Vienna and the University of Vienna, Vienna, Austria; Lawrence Berkeley National Laboratory

**Keywords:** ammonia and oxygen limitation, ammonia-oxidizing bacteria, chemostat, nitrification, *Nitrosomonas europaea*, transcriptome

## Abstract

Ammonia-oxidizing microorganisms perform the first step of nitrification, the oxidation of ammonia to nitrite. The bacterium Nitrosomonas europaea is the best-characterized ammonia oxidizer to date. Exposure to hypoxic conditions has a profound effect on the physiology of *N. europaea*, e.g., by inducing nitrifier denitrification, resulting in increased nitric and nitrous oxide production. This metabolic shift is of major significance in agricultural soils, as it contributes to fertilizer loss and global climate change. Previous studies investigating the effect of oxygen limitation on *N. europaea* have focused on the transcriptional regulation of genes involved in nitrification and nitrifier denitrification. Here, we combine steady-state cultivation with whole-genome transcriptomics to investigate the overall effect of oxygen limitation on *N. europaea*. Under oxygen-limited conditions, growth yield was reduced and ammonia-to-nitrite conversion was not stoichiometric, suggesting the production of nitrogenous gases. However, the transcription of the principal nitric oxide reductase (cNOR) did not change significantly during oxygen-limited growth, while the transcription of the nitrite reductase-encoding gene (*nirK*) was significantly lower. In contrast, both heme-copper-containing cytochrome *c* oxidases encoded by *N. europaea* were upregulated during oxygen-limited growth. Particularly striking was the significant increase in transcription of the B-type heme-copper oxidase, proposed to function as a nitric oxide reductase (sNOR) in ammonia-oxidizing bacteria. In the context of previous physiological studies, as well as the evolutionary placement of *N. europaea’s* sNOR with regard to other heme-copper oxidases, these results suggest sNOR may function as a high-affinity terminal oxidase in *N. europaea* and other ammonia-oxidizing bacteria.

**IMPORTANCE** Nitrification is a ubiquitous microbially mediated process in the environment and an essential process in engineered systems such as wastewater and drinking water treatment plants. However, nitrification also contributes to fertilizer loss from agricultural environments, increasing the eutrophication of downstream aquatic ecosystems, and produces the greenhouse gas nitrous oxide. As ammonia-oxidizing bacteria are the most dominant ammonia-oxidizing microbes in fertilized agricultural soils, understanding their responses to a variety of environmental conditions is essential for curbing the negative environmental effects of nitrification. Notably, oxygen limitation has been reported to significantly increase nitric oxide and nitrous oxide production during nitrification. Here, we investigate the physiology of the best-characterized ammonia-oxidizing bacterium, Nitrosomonas europaea, growing under oxygen-limited conditions.

## Abstract (precis)

Nitrification is a ubiquitous microbially mediated process in the environment and an essential process in engineered systems such as wastewater and drinking water treatment plants. However, nitrification also contributes to fertilizer loss from agricultural environments, increasing the eutrophication of downstream aquatic ecosystems, and produces the greenhouse gas nitrous oxide. As ammonia-oxidizing bacteria are the most dominant ammonia-oxidizing microbes in fertilized agricultural soils, understanding their responses to a variety of environmental conditions is essential for curbing the negative environmental effects of nitrification. Notably, oxygen limitation has been reported to significantly increase nitric oxide and nitrous oxide production during nitrification. Here, we investigate the physiology of the best-characterized ammonia-oxidizing bacterium, Nitrosomonas europaea, growing under oxygen-limited conditions.

## INTRODUCTION

Nitrification is a microbially mediated aerobic process involving the successive oxidation of ammonia (NH_3_) and nitrite (NO_2_^−^) to nitrate (NO_3_^−^) (1). In oxic environments, complete nitrification is accomplished through the complementary metabolisms of ammonia-oxidizing bacteria (AOB)/archaea (AOA) and nitrite-oxidizing bacteria (NOB) or by comammox bacteria (2, 3). The existence of nitrite-oxidizing archaea (NOA) has been proposed but not yet confirmed (4). Although an essential process during wastewater and drinking water treatment, nitrification is also a major cause of nitrogen (N) loss from N-amended soils. Nitrifiers increase N loss through the production of NO_3_^−^, which is more susceptible to leaching from soils than ammonium (NH_4_^+^), serves as terminal electron acceptor for denitrifiers, and contributes to the eutrophication of downstream aquatic environments (5).

In addition, ammonia oxidizers produce and release nitrogenous gases such as nitric (NO) and nitrous (N_2_O) oxide during NH_3_ oxidation at a wide range of substrate and oxygen (O_2_) concentrations (6, 7). Nitrogenous gases are formed through enzymatic processes (8–13) but also by a multitude of chemical reactions that use the key metabolites of ammonia oxidizers, hydroxylamine (NH_2_OH) and NO_2_^−^ (or its acidic form HNO_2_), as the main precursors (14, 15). AOB, in particular, release NO and N_2_O either during NH_2_OH oxidation (16–21) or via nitrifier denitrification—the reduction of NO_2_^−^ to N_2_O via NO (22–25). The first pathway is the dominant process at atmospheric O_2_ levels, while the latter is more important under O_2_-limited (hypoxic) conditions (26, 27), where NO_2_^−^ and NO serve as alternative sinks for electrons generated by NH_3_ oxidation.

**FIG 1:**
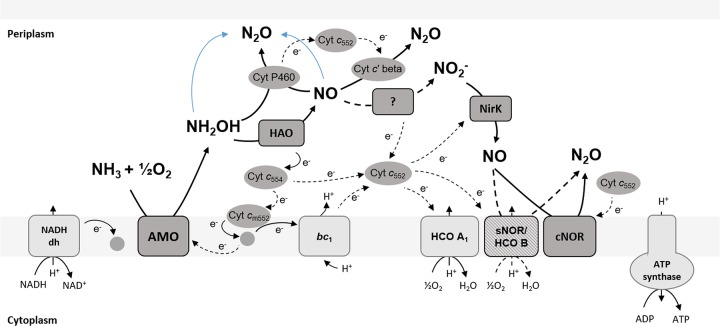
A simplified schematic of electron transport and NO/N_2_O-producing pathways in *N. europaea*. Solid lines indicate confirmed and dashed lines indicate postulated reactions or electron transfer processes. Abiotic N_2_O production is indicated in blue. NADH dh, NADH dehydrogenase (complex I); AMO, ammonia monooxygenase; HAO, hydroxylamine dehydrogenase; NirK, nitrite reductase; *bc*_1_, cytrochrome *bc-*I complex (complex III); HCO A1, heme-copper-containing *cytochrome c* oxidase A1-type (complex IV); sNOR/HCO B, heme-copper-containing NO reductase/heme-copper-containing cytochrome *c* oxidase B-type (complex IV); cNOR, heme-iron-containing nitric oxide reductase.

Nitrosomonas europaea strain ATCC 19718 was the first AOB to have its genome sequenced (28) and is widely used as a model organism in physiological studies of NH_3_ oxidation and NO/N_2_O production in AOB (27, 29–36). The enzymatic background of NO and N_2_O production in *N. europaea* is complex and involves multiple interconnected processes (Fig. 1). Most AOB harbor a copper-containing nitrite reductase, NirK, which is necessary for efficient NH_3_ oxidation by *N. europaea* at atmospheric O_2_ levels. NirK is also involved in but not essential for NO production during nitrifier denitrification in *N. europaea* (26, 27, 29, 35) and is upregulated in response to high NO_2_^−^ concentrations (37). Moreover, two forms of membrane-bound cytochrome (cyt) *c* oxidases (cNOR and sNOR) and three cytochromes, referred to as cyt P460 (CytL), cyt *c′* beta (CytS), and cyt *c*_554_ (CycA), have been implicated in N_2_O production in *N. europaea* and other AOB (12, 24, 32, 38–40). However, the involvement of cyt *c*_554_ in N_2_O production has recently been disputed (41). Finally, recent research has confirmed that the oxidation of NH_3_ to NO_2_^−^ in AOB includes the formation of NO as an obligate intermediate, produced by NH_2_OH oxidation via the hydroxylamine dehydrogenase (HAO) (20). The enzyme responsible for the oxidation of NO to NO_2_^−^ (the proposed nitric oxide oxidase) has not yet been identified (40).

The production of NO and N_2_O by *N. europaea*, grown under oxic as well as hypoxic (oxygen-limited) conditions, was previously demonstrated and quantified in multiple batch and chemostat culture studies (11, 12, 34, 35, 42, 43). Furthermore, recent studies have investigated the instantaneous rate of NO and N_2_O production by *N. europaea* during the transition from oxic to oxygen-limited or anoxic conditions (12, 35, 36). Despite this large body of literature describing the effect of oxygen (O_2_) limitation on NH_3_ oxidation and NO/N_2_O production in *N. europaea*, little attention has been paid to the regulation of other processes under these conditions. Previous studies have utilized reverse transcription-quantitative PCR (RT-qPCR) assays to examine transcriptional patterns of specific mainly N cycle-related genes in AOB grown under O_2_-limited conditions (34, 36, 44). To date, no study has evaluated the global transcriptomic response of *N. europaea* to O_2_-limited growth. However, research on the effect of stressors other than reduced O_2_ tension have demonstrated the suitability of transcriptomics for the analysis of physiological responses in AOB (43, 45–48).

*N. europaea* utilizes the Calvin-Benson-Bassham (CBB) cycle to fix inorganic carbon (28, 49). Whereas all genome-sequenced AOB appear to use the CBB cycle, differences exist in the number of copies of ribulose-1,5-bisphosphate carboxylase/oxygenase (RuBisCO) genes encoded as well as the presence or absence of carbon dioxide (CO_2_)-concentrating mechanisms (50–52). *N. europaea* harbors a single form IA green-like (high-affinity) RuBisCO enzyme and two carbonic anhydrases but no carboxysome-related genes (28). RuBisCO is considered to function optimally in hypoxic environments, as it also uses O_2_ as a substrate and produces the off-path intermediate 2-phosphoglycolate (53, 54). However, the effects of O_2_ limitation on the transcription of RuBisCO-encoding genes and resulting growth yield in AOB are still poorly understood.

In this study, we expand upon previous work investigating the effects of O_2_ limitation on *N. europaea* by profiling the transcriptomic response to substrate (NH_3_) versus O_2_ limitation. *N. europaea* was grown under steady-state NH_3_- or O_2_-limited conditions, which allowed for the investigation of differences in transcriptional patterns between growth conditions. We observed a downregulation of genes associated with CO_2_ fixation as well as increased expression of two distinct heme-copper-containing cytochrome *c* oxidases (HCOs) during O_2_-limited growth. Our results provide new insights into how *N. europaea* physiologically adapts to thrive in O_2_-limited environments and identified putative key enzymes for future biochemical characterization.

## RESULTS AND DISCUSSION

### Growth characteristics.

**FIG 2:**
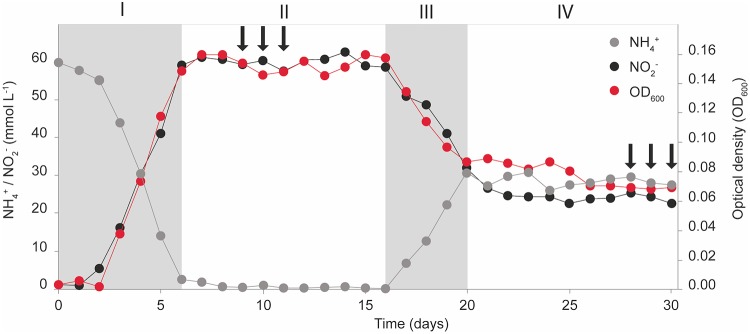
*N. europaea* culture dynamics and sampling scheme. *N. europaea* grown in a chemostat operated in batch mode (I), under steady-state NH_3_-limited conditions as a continuous culture (II), transitioning from NH_3_-limited to O_2_-limited steady-state growth as a continuous culture (III), and under steady-state O_2_-limited conditions as a continuous culture (IV). Arrows indicate transcriptome sampling points during NH_3_-limited (days 9, 10, and 11) and O_2_-limited (days 28, 29, and 30) steady-state growth.

*N. europaea* was grown as a continuous steady-state culture under both NH_3_- and O_2_-limited growth conditions. During NH_3_-limited steady-state growth, the culture was kept oxic with a constant supply of filtered atmospheric air, was continuously stirred (400 rpm), and contained a standing NO_2_^−^ concentration of ∼60 mmol liter^−1^. *N. europaea* grown under NH_3_-limited conditions consumed ∼98% of substrate provided; therefore, cultures were considered to have nonlimiting amounts of O_2_ (Table 1). In contrast, during O_2_-limited steady-state growth, no additional air inflow was provided, but the stirring was increased (800 rpm) to facilitate O_2_ transfer between the headspace and growth medium. As a consequence of O_2_ limitation, the medium contained standing concentrations (∼30 mmol liter^−1^) of both NH_4_^+^ and NO_2_^−^ (Fig. 2; Table 1).

**TABLE 1:** Comparison of *N. europaea* growth characteristics and NH_4_^+^ to NO_2_^−^ conversion stoichiometry during NH_3_- and O_2_-limited steady-state growth

Growth condition	Period (days)	OD_600_^a^	Input NH_3_^b^ (mmol day^−1^)	NH_3_ consumed^a^ (mmol day^−1)^	Steady-state^a^ NH_4_^+^ (mmol liter^−1^)	Steady-state^a^ NO_2_^−^ (mmol liter^−1^)	N balance^a^,^c^,^d^ (mmol)	Ammonia oxidation rate^a^,^d^ (q_NH3_) (mmol g [dry cell weight]^−1^ h^−1^)	Apparent growth yield^a^,^d^ (Y) (g [dry cell weight] mol^−1^ NH_3_)
NH_3_ limited	7–16	0.15 ± 0.01	14.4	14.2 ± 0.1	0.9 ± 0.5	60.1 ± 1.4	61.0 ± 1.7 A	24.04 ± 0.93 C	0.42 ± 0.02 C
	9–11	0.15 ± 0.004	14.4	14.2 ± 0.1	0.9 ± 0.4	59.1 ± 1.4	60.0 ± 1.8 c	24.73 ± 0.53 c	0.40 ± 0.01 c
O_2_ limited growth	23–32	0.07 ± 0.01	14.4	7.5 ± 0.4	28.9 ± 1.5	24.1 ± 0.8	52.8 ± 1.8 B	26.44 ± 2.28 D	0.38 ± 0.03 D
	28–30	0.07 ± 0.0005	14.4	7.5 ± 0.3	28.6 ± 1.1	24.3 ± 1.4	52.9 ± 2.4 d	28.51 ± 1.13 d	0.35 ± 0.01 d

*^a^* Average values from 3 sampling days or 10-day steady-state period, ± standard deviations (see Table S1 in the supplemental material).

*^b^* The NH_4_^+^ concentration of the influx medium (60 mmol liter^−1^) multiplied by the influx rate (0.24 liter day^−1^).

*^c^* Sum of effluent NH_4_^+^ and NO_2_^−^ concentrations.

*^d^* Letters A and B represent highly significant differences (*P* ≤ 0.01), and letters C and D represent significant differences (*P* ≤ 0.05) within parameters. Capital letters represent comparisons between 10-day periods, whereas lowercase letters represent comparisons between 3-day periods.

10.1128/mSystems.00562-19.3 TABLE S1 *N. europaea* culture dynamics and NH_3_ oxidation activity calculations. Shaded areas represent non-steady-state growth phases. Transcriptome sampling days are indicated in bold; g (dry cell weight) in the chemostat; q_NH3_, NH_3_ consumption rate; Y, apparent growth yield. Download Table S1, PDF file, 0.05 MB. Copyright © 2020 Sedlacek et al. 2020 Sedlacek et al. This content is distributed under the terms of the Creative Commons Attribution 4.0 International license.

During NH_3_-limited steady-state growth (days 7 to 16) (Fig. 2), *N. europaea* stoichiometrically oxidized all supplied NH_4_^+^ to NO_2_^−^ (N balance = 61.0 ± 1.7 mmol liter^−1^) and maintained an optical density at 600 nm (OD_600_) of 0.15 ± 0.01 (Table 1). During O_2_-limited steady-state growth (days 23 to 32) (Fig. 2), *N. europaea* was able to consume on average 31.1 ± 1.5 mmol liter^−1^ (51.8%) of the supplied NH_4_^+^ and maintained an OD_600_ of 0.07 ± 0.01 (Table 1). A decrease in OD_600_ was expected, as the O_2_-limited culture oxidized less total substrate (NH_4_^+^), resulting in less biomass produced. The conversion of NH_4_^+^ to NO_2_^−^ was not stoichiometric during O_2_-limited growth, as only 77.5% (24.1 ± 0.8 mmol liter^−1^) of the NH_4_^+^ oxidized was measured as NO_2_^−^ in the effluent, resulting in an N balance of 52.8 ± 1.8 mmol liter^−1^ (Table 1). The significant difference (*P* ≤ 0.01) in the N balance between NH_4_^+^ consumed and NO_2_^−^ formed during O_2_-limited growth is in accordance with previous reports and likely due to increased N loss in the form of NH_2_OH, NO, and N_2_O under O_2_-limited conditions (12, 35, 42, 55).

The dilution rate (0.01 h^−1^) of the chemostat was kept constant during both NH_3_- and O_2_-limited growth, and resulted in 14.4 mmol day^−1^ NH_4_^+^ delivered into the chemostat. On days 9, 10, and 11, which were sampled for NH_3_-limited growth transcriptomes, *N. europaea* consumed NH_3_ at a rate (q_NH3_) of 24.73 ± 0.53 mmol g (dry cell weight)^−1^ h^−1^ with an apparent growth yield (Y) of 0.40 ± 0.01 g (dry cell weight) mol^−1^ NH_3_. During days sampled for O_2_-limited growth transcriptomes (days 28, 29, and 30), the q_NH3_ was significantly higher (28.51 ± 1.13 mmol g [dry cell weight]^−1^ h^−1^; *P* ≤ 0.05), while Y was significantly lower (0.35 ± 0.01 g [dry cell weight] mol^−1^ NH_3_; *P* ≤ 0.05). When the whole 10-day NH_3_- and O_2_-limited steady-state growth periods were considered, the q_NH3_ and Y trends remained statistically significant (*P* ≤ 0.05) (Table 1). Overall, NH_3_ oxidation was less efficiently coupled to biomass production under O_2_-limited growth conditions.

### Global transcriptomic response of *N. europaea* to growth under NH_3_- versus O_2_-limited conditions.

**FIG 3:**
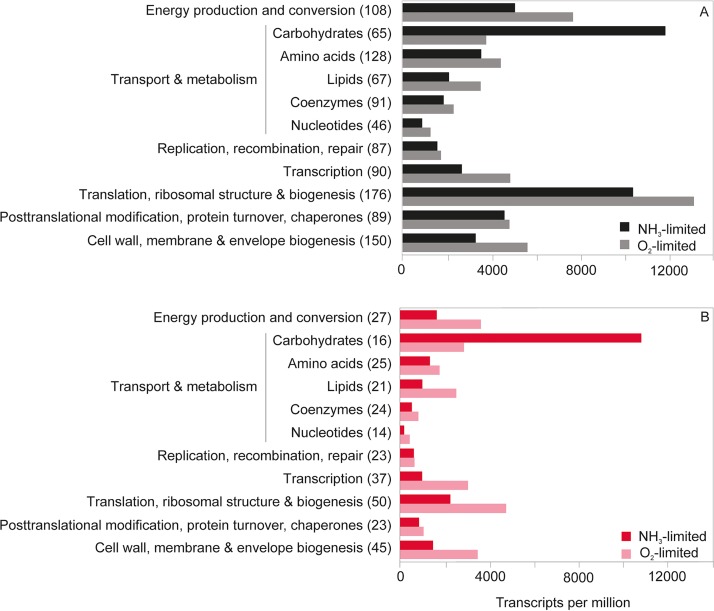
The sum of transcripts per million (TPM) for protein-coding genes transcribed in given COG categories (number of transcribed genes per category is given in parentheses) in the *N. europaea* transcriptomes. (A) Contributions and numbers of all transcribed genes in a given COG category. (B) Contributions and numbers of statistically significantly differentially transcribed genes in a given COG category.

Under both NH_3_- and O_2_-limited growth conditions, transcripts mapping to 2,535 of 2,572 protein-coding genes (98.5%) and 3 RNA-coding genes (*ffs*, *rnpB*, and transfer-messenger RNA [tmRNA]) were detected. Many of the 37 genes not detected encode phage elements or transposases, some of which may have been excised from the genome in the >15 years of culturing since genome sequencing (see Data Set S1 in the supplemental material). In addition, no tRNA transcripts were detected. The high proportion of transcribed genes is in line with recent *N. europaea* transcriptomic studies, where similarly high fractions of transcribed genes were detected (43, 48). A significant difference in transcript levels between growth conditions was detected for 615 (∼24%) of transcribed genes (see Fig. S1). Of these 615 genes, 435 (∼71%) were present at higher levels, while 180 (∼29%) were present at lower levels during O_2_-limited growth. Genes encoding hypothetical proteins with no further functional annotation accounted for ∼21% (130) of the differentially transcribed genes (Data Set S1). Steady-state growth under O_2_-limited conditions mainly impacted the transcription of genes in clusters of orthologous groups (COGs) related to transcription and translation, ribosome structure and biogenesis, carbohydrate transport and metabolism, and energy production and conversion (Fig. 3).

10.1128/mSystems.00562-19.1 FIG S1 Differential transcription (mean TPMs) of *N. europaea* genes during NH_3_- versus O_2_-limited growth. Transcripts differentially regulated by ≥1.5-fold change and a Welch’s *t* test *P* value threshold of ≤0.01 or ≤0.05 are shown in red or black, respectively. Download FIG S1, PDF file, 1.7 MB. Copyright © 2020 Sedlacek et al. 2020 Sedlacek et al. This content is distributed under the terms of the Creative Commons Attribution 4.0 International license.

10.1128/mSystems.00562-19.2 DATA SET S1 Differential transcription (mean TPMs) and the corresponding fold changes of all the *N. europaea* genes that transcripts were detected during NH_3_- and/or O_2_-limited growth. *P* values were determined by a Welch’s *t* test. Download Data Set S1, XLSX file, 0.3 MB. Copyright © 2020 Sedlacek et al. 2020 Sedlacek et al. This content is distributed under the terms of the Creative Commons Attribution 4.0 International license.

### Universal and reactive oxygen stress.

The transcript levels of various chaperone proteins and sigma factors considered to be involved in the general stress response in *N. europaea* (45) differed between NH_3_- and O_2_-limited growth, with no discernible trend of regulation (see Table S2; Data Set S1). Overall, prolonged exposure to O_2_ limitation did not seem to induce a significantly increased general stress response in *N. europaea*. Key genes involved in oxidative stress defense (superoxide dismutase, catalase, peroxidases, and thioredoxins) were transcribed at lower levels during O_2_-limited growth, as expected (Table S2; Data Set S1). Surprisingly, rubredoxin (NE1426) and a glutaredoxin family protein-encoding gene (NE2328) did not follow this trend and were transcribed at significantly higher levels (2.8- and 1.8-fold, respectively) during O_2_-limited growth (Table S2). Although their role in *N. europaea* is currently unresolved, both have been proposed to be involved in cellular oxidative stress response (56, 57), iron homeostasis (58, 59), or both.

10.1128/mSystems.00562-19.4 TABLE S2 Differential expression of select genes during NH_3_-limited versus O_2_-limited growth. *, *P* ≤ 0.05; **, *P* ≤ 0.01. Download Table S2, PDF file, 0.1 MB. Copyright © 2020 Sedlacek et al. 2020 Sedlacek et al. This content is distributed under the terms of the Creative Commons Attribution 4.0 International license.

### Carbon fixation and carbohydrate and storage compound metabolism.

**FIG 4:**
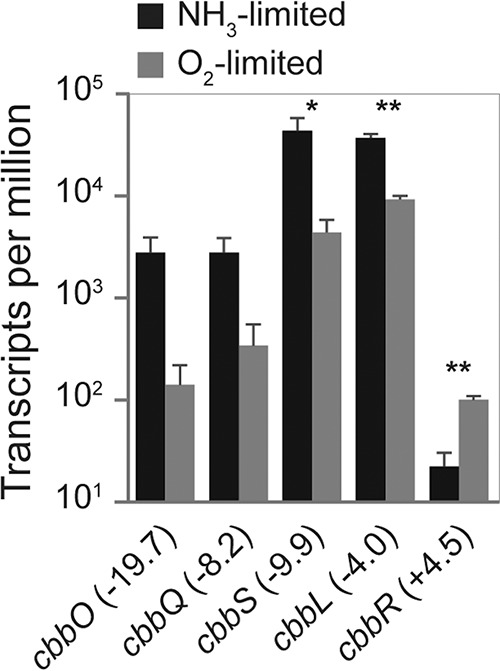
Mean TPMs of all RuBisCO-encoding genes (*cbbOQSL*) and the corresponding transcriptional regulator (*cbbR*) in *N. europaea*. The fold changes of gene transcription between NH_3_- versus O_2_-limited growth are given in parentheses. Error bars represent the standard deviations between replicate samples (*n* = 3) for each growth condition. A Welch’s *t* test was used to determine significantly differentially transcribed genes. *, *P* < 0.05; **, *P* < 0.01. For gene annotations, refer to Table S2 in the supplemental material.

There was a particularly strong effect of O_2_-limited growth on the transcription of several genes related to CO_2_ fixation (Fig. 3B). The four genes of the RuBisCO-encoding *cbb* operon (*cbbOQSL*) were among the genes displaying the largest decrease in detected transcript numbers (Fig. 4; Table S2). Correspondingly, the transcriptional repressor of the *cbb* operon (*cbbR*) was transcribed at 4.5-fold higher levels (Fig. 4; Table S2). This agrees with the previously reported decrease in transcription of the *N. europaea cbbOQSL* operon in O_2_-limited batch culture experiments (60). The reduced transcription of RuBisCO-encoding genes potentially reflects a decreased RuBisCO enzyme concentration needed to maintain an equivalent CO_2_ fixation rate during O_2_-limited growth. Since O_2_ acts as a competing substrate for the RuBisCO active site, the CO_2_-fixing carboxylase reaction proceeds more efficiently at lower O_2_ concentrations (53, 61, 62). When *N. europaea* is grown under CO_2_ limitation, the transcription of RuBisCO-encoding genes increases significantly (43, 60, 63). Due to the absence of carboxysomes, *N. europaea* appears to regulate CO_2_ fixation at the level of RuBisCO enzyme concentration.

Genes encoding the remaining enzymes of the CBB pathway and carbonic anhydrases were not significantly differentially regulated, with the exception of the transketolase-encoding *cbbT* gene (Table S2). Likewise, almost no differences in transcription were observed for the majority of genes in other central metabolic pathways (glycolysis/gluconeogenesis, tricarboxylic acid [TCA] cycle) (Data Set S1). As the specific growth rate of *N. europaea* was kept constant during both NH_3_- and O_2_-limited growth, it is not surprising that genes associated with these core catabolic pathways were transcribed at comparable levels.

Differential transcription of polyphosphate (PP) metabolism-related genes suggests an increased accumulation of PP storage during O_2_-limited growth. Transcripts of the polyphosphate kinase (*ppk*) involved in PP synthesis were detected in significantly higher numbers (2.1-fold), while transcription of the gene encoding the PP-degrading exopolyphosphatase (*ppx*) did not change (Table S2). Indeed, *N. europaea* was previously shown to accumulate PP when ATP generation (NH_3_ oxidation) and ATP consumption become uncoupled and surplus ATP is available (64). As the specific growth rate was kept constant throughout the experiment, PP accumulation could be a result of increased efficiency in ATP-consuming pathways, such as CO_2_ fixation or oxidative stress-induced repair. A decrease in the reaction flux through the energetically wasteful oxygenase reaction catalyzed by RuBisCO could result in surplus ATP being diverted to PP production.

### Energy conservation.

Genes encoding the known core enzymes of the NH_3_ oxidation pathway in *N. europaea* were all highly transcribed during both NH_3_- and O_2_-limited growth (Table S2). These included ammonia monooxygenase (AMO; *amoCAB* operons and the singleton *amoC* gene) and the genes encoding HAO (*haoBA*) and the accessory cyt *c*_554_ (*cycA*) and cyt *c*_m552_ (*cycX*). Due to a high level of sequence conservation among the multiple AMO and HAO operons (65), it is not possible to decipher the transcriptional responses of paralogous genes in these clusters. Therefore, we report the regulation of AMO and HAO operons as single units (Table S2). The transcript numbers of genes in the AMO operons decreased up to 3.3-fold during O_2_-limited growth, while transcripts of the singleton *amoC* were present at 1.9-fold higher levels. However, these transcriptional differences were not statistically significant. The HAO cluster genes were also not significantly differentially transcribed (Table S2).

Previous research has shown that transcription of AMO, and to a lesser extent of HAO, is induced by NH_3_ in a concentration-dependent manner (66). In contrast, other studies have reported an increase in *amoA* transcription by *N. europaea* following substrate limitation (44, 67). Furthermore, *N. europaea* has been reported to increase *amoA* and *haoA* transcription during growth under low-O_2_ conditions (34). However, exposure to repeated transient anoxia did not significantly change *amoA* or *haoA* mRNA levels (36). As both NH_3_ and O_2_ limitation were previously shown to induce transcription of AMO- and HAO-encoding genes, the high transcription levels observed here under both NH_3_- and O_2_-limited steady-state growth conditions are not surprising.

The periplasmic red copper protein nitrosocyanin (NcyA) was among the most highly transcribed genes under both NH_3_- and O_2_-limited growth conditions (Table S2). Nitrosocyanin has been shown to be expressed at levels similar to those of other nitrification and electron transport proteins (68) and is among the most abundant proteins commonly found in AOB proteomes (47, 69). To date, the nitrosocyanin-encoding gene *ncyA* has been identified only in AOB genomes (24) and has been proposed as a candidate for the nitric oxide oxidase (40). However, as comammox *Nitrospira* do not encode *ncyA* (2, 3, 13), nor do all genome-sequenced AOB (70), nitrosocyanin cannot be the NO oxidase in all ammonia oxidizers. In this study, a slight (1.7-fold) but not statistically significantly higher number of *ncyA* transcripts was detected during O_2_-limited growth (Table S2). This agrees with a previous study comparing *ncyA* mRNA levels in *N. europaea* continuous cultures grown under high- and low-O_2_ conditions (44). However, *N. europaea* performing pyruvate-dependent NO_2_^−^ reduction also significantly upregulated *ncyA*, while transcription of *amoA* and *haoA* decreased (44). Overall, there is evidence for an important role of nitrosocyanin in NH_3_ oxidation or electron transport in AOB, but further experiments are needed to elucidate its exact function.

Three additional cytochromes are considered to be involved in the ammonia-oxidizing pathway of *N. europaea*: (i) cyt *c*_552_ (*cycB*), essential for electron transfer; (ii) cyt P460 (*cytL*), responsible for N_2_O production from NO and hydroxylamine (39); and (iii) cyt *c*′-beta (*cytS*), hypothesized to be involved in N oxide detoxification and metabolism (24, 71). All three were among the most highly transcribed genes (top 20%) under both growth conditions (Table S2). In this study, *cytS* was transcribed at significantly lower levels (2.3-fold) during O_2_-limited growth. However, transcription levels of *cycB* and *cytL* were not significantly different (Table S2). While the *in vivo* function of *cytS* remains elusive, it is important to note that in contrast to *ncyA*, the *cytS* gene is present in all sequenced AOB and comammox *Nitrospira* genomes (12, 13, 52). The ubiquitous detection of *cytS* in genomes of all AOB, comammox *Nitrospira*, and in methane-oxidizing bacteria capable of NH_3_ oxidation (72) indicates that cyt *c*′-beta might play an important yet unresolved role in bacterial aerobic NH_3_ oxidation.

### Nitrifier denitrification.

**FIG 5:**
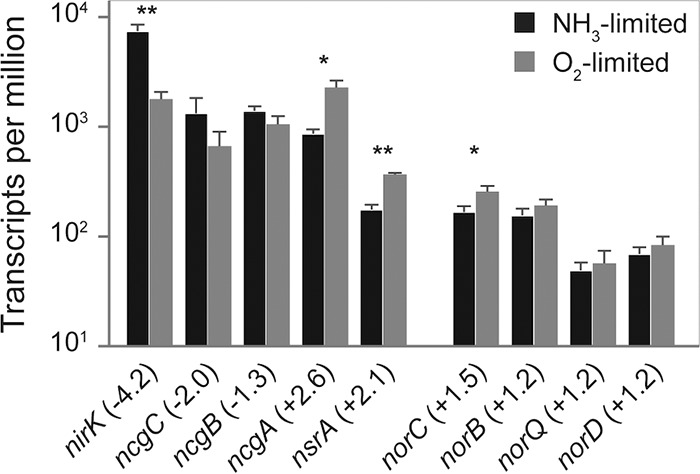
Mean TPMs of genes encoding the NirK and cNOR gene clusters in *N. europaea*. The fold changes of gene transcription between NH_3_- versus O_2_-limited growth are given in parentheses. Error bars represent the standard deviations between replicate samples (*n* = 3) for each growth condition. A Welch’s *t* test was used to determine significantly differentially transcribed genes. *, *P* < 0.05; **, *P* < 0.01. For gene annotations refer to Table S2.

During O_2_-limited growth, *N. europaea* either performs nitrifier denitrification or experiences a greater loss of N intermediates such as NH_2_OH (73) or NO (20), which leads to the observed N imbalance between total NH_4_^+^ consumed and NO_2_^−^ produced (Fig. 2; Table 1). The Cu-containing NO_2_^−^ reductase NirK and the iron-containing membrane-bound cyt *c*-dependent NO reductase (cNOR; NorBC) are considered to be the main nitrifier denitrification enzymes (24, 35). *N. europaea* NirK plays an important role in both nitrifier denitrification and NH_3_ oxidation (27) and is known to be expressed during both O_2_-replete and -limited growth (29, 30, 35). However, under O_2_-limited conditions, *nirK* was among the genes with the largest decrease in transcript numbers (4.2-fold) observed in this study (Fig. 5; Table S2). In *N. europaea*, *nirK* transcription is regulated via the nitrite-sensitive transcriptional repressor *nsrA* (30). Thus, in contrast to the *nirK* of many denitrifiers (74), *nirK* transcription in *N. europaea* is regulated in response to NO_2_^−^ concentration and not NO or O_2_ availability (31, 34, 48). The reduced O_2_ supply during O_2_-limited growth resulted in an ∼50% decrease in total NH_3_ oxidized and an ∼60% reduction in steady-state NO_2_^−^ concentration (Fig. 2; Table 1). The decrease in NO_2_^−^ concentration during O_2_-limited growth likely induced the transcription of *nsrA*, which was significantly (2.1-fold) upregulated (Fig. 5; Table S2). Therefore, the large decrease in *nirK* transcription observed here was likely due to the lower NO_2_^−^ concentrations and not a direct reflection of overall nitrifier denitrification activity. In more natural nitrifying systems (e.g., agricultural soils or wastewater treatment plants [WWTPs]) changes in NO_2_^−^ concentration could have a greater effect on AOB *nirK* expression than O_2_ availability. However, it should be noted that environmental NO_2_^−^ concentrations are unlikely to reach those observed in this study (30 to 60 mmol liter^−1^ NO_2_^−^).

Regulation of *nirK* transcription in response to primarily NO_2_^−^ and not O_2_ concentration is consistent with the observation that NirK is not essential for NO_2_^−^ reduction to NO in *N. europaea*. This supports the hypothesis that a not-yet-identified nitrite reductase is present in this organism. Previously, it was shown that *N. europaea nirK* knockout mutants are still able to enzymatically produce NO and N_2_O (29, 35), even if hydrazine is oxidized by HAO instead of hydroxylamine as an electron donor (35). In addition, NO and N_2_O formation have also been observed in the AOB Nitrosomonas communis that does not encode *nirK* (12). The other three genes in the NirK cluster (*ncgCBA*) were differentially transcribed, with *ncgC* and *ncgB* being transcribed at lower levels (2- and 1.3-fold, respectively), while *ncgA* was transcribed at a significantly higher level (2.6-fold) during O_2_-limited growth. The role of *ncgCBA* in *N. europaea* has not been fully elucidated, but all three genes were previously implicated in the metabolism or tolerance of N oxides and NO_2_^−^ (31).

**FIG 6:**
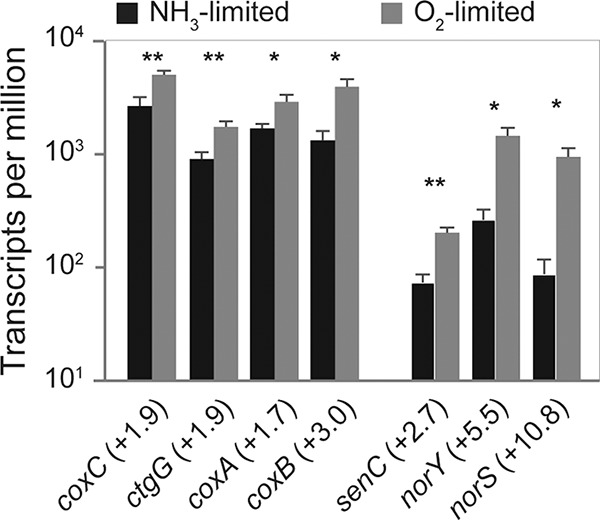
Mean TPMs of all genes encoding the A1-type and B-type HCO in *N. europaea*. The fold changes of gene transcription between NH_3_- versus O_2_-limited growth are given in parentheses. Error bars represent the standard deviations between replicate samples (*n* = 3) for each growth condition. A Welch’s *t* test was used to determine significantly differentially transcribed genes. *, *P* < 0.05; **, *P* < 0.01. For gene annotations, refer to Table S2.

In contrast, transcripts of the *norCBQD* gene cluster, encoding the iron-containing cyt *c*-dependent cNOR-type NO reductase, were present at slightly higher (1.2- to 1.5-fold) but not significantly different levels during O_2_-limited growth (Fig. 5; Table S2). Previous research has demonstrated that in *N. europaea*, cNOR functions as the main NO reductase under anoxic and hypoxic conditions (35). Interestingly, all components of the proposed alternative heme-copper-containing NO reductase (sNOR), including the NO/low-oxygen sensor *senC* (24), were transcribed at significantly higher levels (2.7- to 10.8-fold) during O_2_-limited growth (Fig. 6; Table S2). Therefore, it is possible that the phenotype describing cNOR as the main NO reductase in *N. europaea* (35) was a product of short incubation times and that during longer term O_2_-limited conditions, sNOR contributes to NO reduction during nitrifier denitrification. Another possibility is that the increased transcription of sNOR observed here during O_2_-limited growth is primarily related to respiration and not NO reductase activity.

### Respiratory chain and terminal oxidases.

*N. europaea* harbors a low-affinity cyt *c aa*_3_ (A1 type) HCO but not a high-affinity *cbb*_3_-type (C type) cyt *c* HCO harbored by other AOB such as *N. eutropha* or *Nitrosomonas* sp. GH22 (28, 50, 52). Significantly higher numbers of transcripts (1.7- to 3.0-fold) of all three subunits of the cyt *c aa*_3_ HCO and the cyt *c* oxidase assembly gene *ctaG* were detected during O_2_-limited growth (Fig. 6; Table S2). Increased transcription of the terminal oxidase was expected, as it is a common bacterial response to O_2_ limitation (75). In addition, transcripts of all three subunits of the proton translocating cyt *bc*-I complex (complex III) were present in higher numbers (Table S2). The genes encoding NADPH dehydrogenase (complex I) and ATP synthase (complex V) were transcribed at similar levels during both growth conditions (Table S2).

As mentioned above, transcripts of both subunits of sNOR (*norSY*, previously called *coxB_2_A_2_*), and the NO/low-oxygen sensor *senC* were present at significantly higher numbers (2.7- to 10.8-fold) during O_2_-limited growth (Fig. 6; Table S2). The NO reductase function of the sNOR enzyme complex was proposed based on domain similarities between NorY and NorB (24, 32). Yet, *norY* phylogenetically affiliates with and structurally resembles B-type HCOs (76). In addition, NorY does not contain the five well-conserved and functionally important NorB glutamate residues (77), which are present in the canonical NorB of *N. europaea*. All HCOs studied thus far can reduce O_2_ to H_2_O and couple this reaction to proton translocation, albeit B- and C-type HCOs translocate fewer protons per mole O_2_ reduced than A-type HCOs (78). Notably, NO reduction to N_2_O is a known side reaction of the A2-, B-, and C-type but not A1-type HCOs (79–81). The transcriptional induction of sNOR during O_2_-limited growth reported here, as well as the high O_2_ affinity of previously studied B-type HCOs (82), indicates that sNOR might function as a high-affinity terminal oxidase in *N. europaea* and possibly other sNOR-harboring AOB. Furthermore, functionally characterized B-type HCOs display a lower NO turnover rate than the more widespread high-affinity C-type HCOs (79, 80). Taken together, these observations indicate that B-type HCOs, such as sNOR, are ideal for scavenging O_2_ during O_2_-limited growth conditions that coincide with elevated NO concentrations, which would impart a fitness advantage for AOB growing under these conditions. Lastly, the NOR of Roseobacter denitrificans structurally resembles cNOR but contains an HCO-like heme-copper center in place of the heme-iron center of canonical cNORs. Interestingly, this cNOR readily reduces O_2_ to H_2_O but displays very low NO reductase activity (83, 84). Therefore, in line with previous hypotheses (79, 83), the presence of a heme-copper center in NOR/HCO superfamily enzymes, such as the sNOR of *N. europaea*, may indicate O_2_ reduction as the primary enzymatic function. Notably, a recent study provided the first indirect evidence of NO reductase activity of sNOR in the marine NOB, Nitrococcus mobilis (85). However, further research is needed to resolve the primary function of sNOR in nitrifying microorganisms.

### Conclusions.

In this study, we examined the transcriptional response of *N. europaea* to continuous growth under steady-state NH_3_- and O_2_-limited conditions. Overall, O_2_-limited growth resulted in a decreased growth yield but did not invoke a significant stress response in *N. europaea*. On the contrary, a reduced need for oxidative stress defense was evident. Interestingly, no clear differential regulation was observed for genes classically considered to be involved in aerobic NH_3_ oxidation. In contrast, a strong decrease in transcription of RuBisCO-encoding genes during O_2_-limited growth was observed, suggesting that control of CO_2_ fixation in *N. europaea* is exerted at the level of RuBisCO enzyme concentration. Furthermore, the remarkably strong increase in transcription of the genes encoding sNOR (B-type HCO) indicates this enzyme complex might function as a high-affinity terminal oxidase in *N. europaea* and other AOB. Overall, despite lower growth yield, *N. europaea* successfully adapts to growth under hypoxic conditions by regulating core components of its carbon fixation and respiration machinery.

## MATERIALS AND METHODS

### Cultivation.

*N. europaea* ATCC 19718 was cultivated at 30°C as a batch and continuous chemostat culture as previously described (43, 48). Briefly, *N. europaea* was grown in mineral medium containing 30 mmol liter^−1^ (NH_4_)_2_SO_4_, 0.75 mmol liter^−1^ MgSO_4_, 0.1 mmol liter^−1^ CaCl_2_, and trace minerals (10 μmol liter^−1^ FeCl_3_, 1.0 μmol liter^−1^ CuSO_4_, 0.6 μmol liter^−1^ Na_2_Mo_4_O_4_, 1.59 μmol liter^−1^ MnCl_2_, 0.6 μmol liter^−1^ CoCl_2_, 0.096 μmol liter^−1^ ZnCl_2_). After sterilization by autoclaving, the medium was buffered by the addition of 6 ml liter^−1^ autoclaved phosphate-carbonate buffer solution (0.52 mmol liter^−1^ NaH_2_PO_4_·H_2_O, 3.5 mmol liter^−1^ KH_2_PO_4_, 0.28 mmol liter^−1^ Na_2_CO_3_, pH adjusted to 7.0 with HCl).

For steady-state growth, a flowthrough bioreactor (Applikon Biotechnology) with a 1-liter working volume was inoculated with 2% (vol/vol) of an exponential-phase *N. europaea* batch culture. The bioreactor was set to “batch” mode until the NH_4_^+^ concentration reached <5 mmol liter^−1^ (6 days) (see Table S1 in the supplemental material). Subsequently, the bioreactor was switched to continuous flow “chemostat” mode, at a dilution rate/specific growth rate (μ) of 0.01 h^−1^ (doubling time = ∼70 h), which was controlled by a peristaltic pump (Thermo Scientific). The culture was continuously stirred at 400 rpm, and the pH was automatically maintained at 7.0 ± 0.1 by addition of sterile 0.94 mol liter^−1^ (10% [wt/vol]) Na_2_CO_3_ solution. Sterile-filtered (0.2 μm) air, at a rate of 40 ml min^−1^, was supplied during batch and NH_3_-limited steady-state growth. Once NH_3_-limited steady-state was reached (day 7), the chemostat was continuously operated under NH_3_-limited conditions for 10 days. To transition to O_2_-limited steady-state growth, after day 16, the air input was stopped, and the stirring speed was increased to 800 rpm to facilitate gas exchange between the medium and the headspace. The headspace was continuously replenished with O_2_ by the passive diffusion of atmospheric air into the chemostat through open air inlets containing a sterile filter (0.2 μm). O_2_-limited steady-state growth was achieved on day 23 as defined by the persistence of 26.4 to 31 mmol liter^−1^ NH_4_^+^ and the accumulation of 22.8 to 25.5 mmol liter^−1^ NO_2_^−^ in the growth medium. The culture was continuously grown under these conditions for 10 days.

Sterile samples (∼5 ml) were taken on a daily basis. Culture purity was assessed by periodically inoculating ∼100 μl of culture onto lysogeny broth (Sigma-Aldrich) agar plates, which were incubated at 30°C for at least 4 days. Any observed growth on agar plates was considered contamination, and those cultures were discarded. NH_4_^+^ and NO_2_^−^ concentrations were determined colorimetrically (86), and cell density was determined spectrophotometrically (Beckman) by making optical density measurements at 600 nm (OD_600_) (Table S1). Total biomass in grams (dry cell weight) per liter, substrate consumption rate (q_NH3_), and apparent growth yield (Y) were calculated as described in Mellbye et al. (43). To test for statistically significant differences in NH_4_^+^ to NO_2_^−^ conversion stoichiometry, q_NH3_, and Y between NH_3_- and O_2_-limited steady-state growth, a Welch’s *t* test was performed.

### RNA extraction and transcriptome sequencing.

For RNA extraction and transcriptome sequencing, three replicate samples (40 ml) were collected on three separate days during NH_3_-limited (days 9, 10, 11) and O_2_-limited (days 28, 29, 30) steady-state growth (Fig. 2). The samples were harvested by centrifugation (12,400 × *g*, 30 min, 4°C), resuspended in RNeasy RLT buffer with 2-mercaptoethanol, and lysed with an ultrasonication probe (3.5 output, pulse of 30 s on/30 s off for 1 min; Heatsystems Ultrasonic Processor XL). RNA was extracted using the RNeasy minikit (Qiagen) followed by the MICROBExpress-bacteria RNA Enrichment kit (Ambion/Life Technologies) according to the manufacturer’s instructions. Depleted RNA quality was assessed using the Bioanalyzer 6000 Nano Lab-Chip kit (Agilent Technologies). Sequencing libraries were constructed from at least 200 ng rRNA-depleted RNA with the TruSeq targeted RNA expression kit (Illumina), and 100-bp paired-end libraries were sequenced on a HiSeq 2000 (Illumina) at the Center for Genome Research and Biocomputing Core Laboratories (CGRB) at Oregon State University.

### Transcriptome analysis.

Paired-end transcriptome sequence reads were processed and mapped to open reading frames (ORFs) deposited at NCBI for the *N. europaea* ATCC 19718 (NC_004757.1) reference genome using the CLC Genomics Workbench (CLC bio) under default parameters as previously described (43). Residual reads mapping to the rRNA operon were excluded prior to further analysis. An additive consensus read count was manually generated for all paralogous genes. Thereafter, mapped read counts for each gene were normalized to the gene length in kilobases, and the resulting read per kilobase (RPK) values were converted to transcripts per million (TPM) (87). To test for statistically significant differences between transcriptomes obtained from NH_3_- and O_2_-limited steady-state growth, TPMs of biological triplicate samples were used to calculate *P* values based on a Welch’s *t* test. The more stringent Welch’s rather than the Student’s *t* test was selected due to the limited number of biological replicates (88). Additionally, linear fold changes between average TPMs under both growth conditions for each expressed ORF were calculated. Transcripts with a *P* value of ≤0.05 and a transcription fold change of ≥1.5× between conditions were considered present at significantly different levels.

### Data availability.

All retrieved transcriptome sequence data have been deposited in the European Nucleotide Archive (ENA) under the project accession number PRJEB31097.

## References

[1] Kuypers MMM, Marchant HK, Kartal B 2018 The microbial nitrogen-cycling network. Nat Rev Microbiol 16:263–276. doi:10.1038/nrmicro.2018.9. 29398704

[2] Daims H, Lebedeva EV, Pjevac P, Han P, Herbold C, Albertsen M, Jehmlich N, Palatinszky M, Vierheilig J, Bulaev A, Kirkegaard RH, von Bergen M, Rattei T, Bendinger B, Nielsen PH, Wagner M 2015 Complete nitrification by *Nitrospira* bacteria. Nature 528:504–506. doi:10.1038/nature16461. 26610024 PMC5152751

[3] van Kessel M, Speth DR, Albertsen M, Nielsen PH, den Camp HJO, Kartal B, Jetten MSM, Lücker S 2015 Complete nitrification by a single microorganism. Nature 528:555–559. doi:10.1038/nature16459. 26610025 PMC4878690

[4] Kitzinger K, Koch H, Lücker S, Sedlacek CJ, Herbold C, Schwarz J, Daebeler A, Mueller AJ, Lukumbuzya M, Romano S, Leisch N, Karst SM, Kirkegaard R, Albertsen M, Nielsen PH, Wagner M, Daims H 2018 Characterization of the first “*Candidatus* Nitrotoga” isolate reveals metabolic versatility and separate evolution of widespread nitrite-oxidizing bacteria. mBio 9:e01186-18. doi:10.1128/mBio.01186-18. 29991589 PMC6050957

[5] Galloway JN, Townsend AR, Erisman JW, Bekunda M, Cai Z, Freney JR, Martinelli LA, Seitzinger SP, Sutton MA 2008 Transformation of the nitrogen cycle: recent trends, questions, and potential solutions. Science 320:889–892. doi:10.1126/science.1136674. 18487183

[6] Dundee L, Hopkins DW 2001 Different sensitivities to oxygen of nitrous oxide production by *Nitrosomonas europaea* and *Nitrosolobus multiformis*. Soil Biol Biochem 33:1563–1565. doi:10.1016/S0038-0717(01)00059-1.

[7] Shaw LJ, Nicol GW, Smith Z, Fear J, Prosser JI, Baggs EM 2006 *Nitrosospira* spp. can produce nitrous oxide via a nitrifier denitrification pathway. Environ Microbiol 8:214–222. doi:10.1111/j.1462-2920.2005.00882.x. 16423010

[8] Ahn JH, Kwan T, Chandran K 2011 Comparison of partial and full nitrification processes applied for treating high-strength nitrogen wastewaters: microbial ecology through nitrous oxide production. Environ Sci Technol 45:2734–2740. doi:10.1021/es103534g. 21388173

[9] Kool DM, Dolfing J, Wrage N, Van Groenigen JW 2011 Nitrifier denitrification as a distinct and significant source of nitrous oxide from soil. Soil Biol Biochem 43:174–178. doi:10.1016/j.soilbio.2010.09.030.

[10] Santoro AE, Buchwald C, McIlvin MR, Casciotti KL 2011 Isotopic signature of N_2_O produced by marine ammonia-oxidizing archaea. Science 333:1282–1285. doi:10.1126/science.1208239. 21798895

[11] Stein LY 2011 Surveying N_2_O-producing pathways in bacteria, p 131–152. *In* Klotz MG (ed), Methods in enzymology. Academic Press, Waltham, MA. 10.1016/B978-0-12-381294-0.00006-7 21185434

[12] Kozlowski JA, Kits KD, Stein LY 2016 Comparison of nitrogen oxide metabolism among diverse ammonia-oxidizing bacteria. Front Microbiol 7:1090. doi:10.3389/fmicb.2016.01090. 27462312 PMC4940428

[13] Kits KD, Jung MY, Vierheilig J, Pjevac P, Sedlacek CJ, Liu S, Herbold C, Stein LY, Richter A, Wissel H, Brüggemann N, Wagner M, Daims H 2019 Low yield and abiotic origin of N_2_O formed by the complete nitrifier *Nitrospira inopinata*. Nat Commun 10:1836. doi:10.1038/s41467-019-09790-x. 31015413 PMC6478695

[14] Schreiber F, Wunderlin P, Udert KM, Wells GF 2012 Nitric oxide and nitrous oxide turnover in natural and engineered microbial communities: biological pathways, chemical reactions, and novel technologies. Front Microbiol 3:372. doi:10.3389/fmicb.2012.00372. 23109930 PMC3478589

[15] Heil J, Vereecken H, Brüggemann N 2016 A review of chemical reactions of nitrification intermediates and their role in nitrogen cycling and nitrogen trace gas formation in soil. Eur J Soil Sci 67:23–39. doi:10.1111/ejss.12306.

[16] Hooper AB 1968 A nitrite-reducing enzyme from *Nitrosomonas europaea*. Preliminary characterization with hydroxylamine as electron donor. Biochim Biophys Acta 162:49–65. doi:10.1016/0005-2728(68)90213-2. 4298926

[17] Hooper AB, Terry KR 1977 Hydroxylamine oxidoreductase from *Nitrosomonas*: inactivation by hydrogen peroxide. Biochemistry 16:455–459. doi:10.1021/bi00622a018. 836796

[18] Hooper AB, Terry KR, Maxwell PC 1977 Hydroxylamine oxidoreductase of *Nitrosomonas*. Oxidation of diethyldithiocarbamate concomitant with stimulation of nitrite synthesis. Biochim Biophys Acta 462:141–152. doi:10.1016/0005-2728(77)90196-7. 199251

[19] Anderson IC, Poth M, Homstead J, Burdige D 1993 A comparison of NO and N_2_O production by the autotrophic nitrifier *Nitrosomonas europaea* and the heterotrophic nitrifier *Alcaligenes faecalis*. Appl Environ Microbiol 59:3525–3533. 8285659 10.1128/aem.59.11.3525-3533.1993 PMC182494

[20] Caranto JD, Lancaster KM 2017 Nitric oxide is an obligate bacterial nitrification intermediate produced by hydroxylamine oxidoreductase. Proc Natl Acad Sci U S A 114:8217–8222. doi:10.1073/pnas.1704504114. 28716929 PMC5547625

[21] Mellbye BL, Giguere AT, Murthy GS, Bottomley PJ, Sayavedra-Soto LA, Chaplen FWR 2018 Genome-scale, constraint-based modeling of nitrogen oxide fluxes during coculture of *Nitrosomonas europaea* and *Nitrobacter winogradskyi*. mSystems 3:e00170-17. doi:10.1128/mSystems.00170-17. 29577088 PMC5864417

[22] Poth M, Focht DD 1985 ^15^N kinetic analysis of N_2_O production by *Nitrosomonas europaea*: an examination of nitrifier denitrification. Appl Environ Microbiol 49:1134–1141. 16346787 10.1128/aem.49.5.1134-1141.1985 PMC238519

[23] Wrage N, Velthof GL, van Beusichem ML, Oenema O 2001 Role of nitrifier denitrification in the production of nitrous oxide. Soil Biol Biochem 33:1723–1732. doi:10.1016/S0038-0717(01)00096-7.

[24] Klotz MG, Stein LY 2008 Nitrifier genomics and evolution of the nitrogen cycle. FEMS Microbiol Lett 278:146–156. doi:10.1111/j.1574-6968.2007.00970.x. 18031536

[25] Giguere AT, Taylor AE, Suwa Y, Myrold DD, Bottomley PJ 2017 Uncoupling of ammonia oxidation from nitrite oxidation: impact upon nitrous oxide production in non-cropped Oregon soils. Soil Biol Biochem 104:30–38. doi:10.1016/j.soilbio.2016.10.011.

[26] Schmidt I, van Spanning RJM, Jetten M 2004 Denitrification and ammonia oxidation by *Nitrosomonas europaea* wild-type, and NirK- and NorB-deficient mutants. Microbiology 150:4107–4114. doi:10.1099/mic.0.27382-0. 15583163

[27] Cantera JJL, Stein LY 2007 Role of nitrite reductase in the ammonia-oxidizing pathway of *Nitrosomonas europaea*. Arch Microbiol 188:349–354. doi:10.1007/s00203-007-0255-4. 17541778

[28] Chain P, Lamerdin J, Larimer F, Regala W, Lao V, Land M, Hauser L, Hooper A, Klotz M, Norton J, Sayavedra-Soto L, Arciero D, Hommes N, Whittaker M, Arp D 2003 Complete genome sequence of the ammonia-oxidizing bacterium and obligate chemolithoautotroph *Nitrosomonas europaea*. J Bacteriol 185:2759–2773. doi:10.1128/jb.185.9.2759-2773.2003. 12700255 PMC154410

[29] Beaumont HJ, Hommes NG, Sayavedra-Soto LA, Arp DJ, Arciero DM, Hooper AB, Westerhoff HV, van Spanning R 2002 Nitrite reductase of *Nitrosomonas europaea* is not essential for production of gaseous nitrogen oxides and confers tolerance to nitrite. J Bacteriol 184:2557–2560. doi:10.1128/jb.184.9.2557-2560.2002. 11948173 PMC134999

[30] Beaumont HJ, Lens SI, Reijnders WN, Westerhoff HV, van Spanning RJ 2004 Expression of nitrite reductase in *Nitrosomonas europaea* involves NsrR, a novel nitrite-sensitive transcription repressor. Mol Microbiol 54:148–158. doi:10.1111/j.1365-2958.2004.04248.x. 15458412

[31] Beaumont HJ, Lens SI, Westerhoff HV, van Spanning RJ 2005 Novel *nirK* cluster genes in *Nitrosomonas europaea* are required for NirK-dependent tolerance to nitrite. J Bacteriol 187:6849–6851. doi:10.1128/JB.187.19.6849-6851.2005. 16166549 PMC1251594

[32] Cho CMH, Yan T, Liu X, Wu L, Zhou J, Stein LY 2006 Transcriptome of a *Nitrosomonas europaea* mutant with a disrupted nitrite reductase gene (*nirK*). Appl Environ Microbiol 72:4450–4454. doi:10.1128/AEM.02958-05. 16751567 PMC1489665

[33] Pellitteri-Hahn MC, Halligan BD, Scalf M, Smith L, Hickey WJ 2011 Quantitative proteomic analysis of the chemolithoautotrophic bacterium *Nitrosomonas europaea*: comparison of growing-and energy-starved cells. J Proteomics 74:411–419. doi:10.1016/j.jprot.2010.12.003. 21172464

[34] Yu R, Chandran K 2010 Strategies of *Nitrosomonas europaea* 19718 to counter low dissolved oxygen and high nitrite concentrations. BMC Microbiol 10:70. doi:10.1186/1471-2180-10-70. 20202220 PMC2844404

[35] Kozlowski JA, Price J, Stein LY 2014 Revision of N_2_O-producing pathways in the ammonia-oxidizing bacterium, *Nitrosomonas europaea* ATCC 19718. Appl Environ Microbiol 80:4930–4935. doi:10.1128/AEM.01061-14. 24907318 PMC4135743

[36] Yu R, Perez-Garcia O, Lu H, Chandran K 2018 *Nitrosomonas europaea* adaptation to anoxic-oxic cycling: insights from transcription analysis, proteomics and metabolic network modeling. Sci Total Environ 615:1566–1573. doi:10.1016/j.scitotenv.2017.09.142. 29055584

[37] Cua LS, Stein LY 2011 Effects of nitrite on ammonia-oxidizing activity and gene regulation in three ammonia-oxidizing bacteria. FEMS Microbiol Lett 319:169–175. doi:10.1111/j.1574-6968.2011.02277.x. 21470297

[38] Upadhyay AK, Hooper AB, Hendrich MP 2006 NO reductase activity of the tetraheme cytochrome *c*_554_ of *Nitrosomonas europaea*. J Am Chem Soc 128:4330–4337. doi:10.1021/ja055183+. 16569009 PMC2806813

[39] Caranto JD, Vilbert AC, Lancaster KM 2016 *Nitrosomonas europaea* cytochrome P460 is a direct link between nitrification and nitrous oxide emission. Proc Natl Acad Sci U S A 113:14704–14709. doi:10.1073/pnas.1611051113. 27856762 PMC5187719

[40] Lancaster KM, Caranto JD, Majer SH, Smith MA 2018 Alternative bioenergy: updates to and challenges in nitrification metalloenzymology. Joule 2:421–441. doi:10.1016/j.joule.2018.01.018.

[41] McGarry JM, Pacheco A 2018 Upon further analysis, neither cytochrome *c*554 from *Nitrosomonas europaea* nor its F156A variant display NO reductase activity, though both proteins bind nitric oxide reversibly. J Biol Inorg Chem 23:861–878. doi:10.1007/s00775-018-1582-4. 29946979

[42] Kester RA, De Boer W, Laanbroek HJ 1997 Production of NO and N_2_O by pure cultures of nitrifying and denitrifying bacteria during changes in aeration. Appl Environ Microbiol 63:3872–3877. 16535707 10.1128/aem.63.10.3872-3877.1997 PMC1389263

[43] Mellbye BL, Giguere A, Chaplen F, Bottomley PJ, Sayavedra-Soto LA 2016 Steady-state growth under inorganic carbon limitation increases energy consumption for maintenance and enhances nitrous oxide production in *Nitrosomonas europaea*. Appl Environ Microbiol 82:3310–3318. doi:10.1128/AEM.00294-16. 27016565 PMC4959225

[44] Beyer S, Gilch S, Meyer O, Schmidt I 2009 Transcription of genes coding for metabolic key functions in *Nitrosomonas europaea* during aerobic and anaerobic growth. J Mol Microbiol Biotechnol 16:187–197. doi:10.1159/000142531. 18594130

[45] Gvakharia BO, Permina EA, Gelfand MS, Bottomley PJ, Sayavedra-Soto LA, Arp DJ 2007 Global transcriptional response of *Nitrosomonas europaea* to chloroform and chloromethane. Appl Environ Microbiol 73:3440–3445. doi:10.1128/AEM.02831-06. 17369330 PMC1907119

[46] Park S, Ely RL 2008 Genome-wide transcriptional responses of *Nitrosomonas europaea* to zinc. Arch Microbiol 189:541–548. doi:10.1007/s00203-007-0341-7. 18097650

[47] Kartal B, Wessels HJ, van der Biezen E, Francoijs KJ, Jetten MS, Klotz MG, Stein LY 2012 Effects of nitrogen dioxide and anoxia on global gene and protein expression in long-term continuous cultures of *Nitrosomonas eutropha* C91. Appl Environ Microbiol 78:4788–4794. doi:10.1128/AEM.00668-12. 22562996 PMC3416356

[48] Pérez J, Buchanan A, Mellbye B, Ferrell R, Chang JH, Chaplen F, Bottomley PJ, Arp DJ, Sayavedra-Soto LA 2015 Interactions of *Nitrosomonas europaea* and *Nitrobacter winogradskyi* grown in co-culture. Arch Microbiol 197:79–89. doi:10.1007/s00203-014-1056-1. 25362506

[49] Sayavedra-Soto LA, Arp DJ 2011 Ammonia-oxidizing *Bacteria*: their biochemistry and molecular biology, p 11–38. *In* Ward BB, Arp DJ, Klotz MG (ed). Nitrification. ASM Press, Washington, DC.

[50] Stein LY, Arp DJ, Berube PM, Chain PS, Hauser L, Jetten MS, Klotz MG, Larimer FW, Norton JM, Op den Camp HJ, Shin M, Wei X 2007 Whole-genome analysis of the ammonia-oxidizing bacterium, *Nitrosomonas eutropha* C91: implications for niche adaptation. Environ Microbiol 9:2993–3007. doi:10.1111/j.1462-2920.2007.01409.x. 17991028

[51] Berg IA 2011 Ecological aspects of the distribution of different autotrophic CO_2_ fixation pathways. Appl Environ Microbiol 77:1925–1936. doi:10.1128/AEM.02473-10. 21216907 PMC3067309

[52] Sedlacek CJ, McGowan B, Suwa Y, Sayavedra-Soto LA, Laanbroek HJ, Stein LY, Norton JM, Klotz MG, Bollmann A 2019 A physiological and genomic comparison of *Nitrosomonas* cluster 6a and 7 ammonia-oxidizing bacteria. Microb Ecol 78:985–994. doi:10.1007/s00248-019-01378-8. 30976841

[53] Andrews TJ, Lorimer GH 1978 Photorespiration—still unavoidable? FEBS Lett 90:1–9. doi:10.1016/0014-5793(78)80286-5.

[54] Badger MR, Bek EJ 2008 Multiple RuBisCo forms in proteobacteria: their functional significance in relation to CO_2_ acquisition by the CBB cycle. J Exp Bot 59:1525–1541. doi:10.1093/jxb/erm297. 18245799

[55] Goreau TJ, Kaplan WA, Wofsy SC, McElroy MB, Valois FW, Watson SW 1980 Production of NO_2_^−^ and N_2_O by nitrifying bacteria at reduced concentrations of oxygen. Appl Environ Microbiol 40:526–532. 16345632 10.1128/aem.40.3.526-532.1980 PMC291617

[56] Prieto-Alamo MJ, Jurado J, Gallardo-Madueno R, Monje-Casas F, Holmgren A, Pueyo C 2000 Transcriptional regulation of glutaredoxin and thioredoxin pathways and related enzymes in response to oxidative stress. J Biol Chem 275:13398–13405. doi:10.1074/jbc.275.18.13398. 10788450

[57] Coulter ED, Kurtz DM Jr 2001 A role for rubredoxin in oxidative stress protection in *Desulfovibrio vulgaris*: catalytic electron transfer to rubrerythrin and two-iron superoxide reductase. Arch Biochem Biophys 394:76–86. doi:10.1006/abbi.2001.2531. 11566030

[58] Andrews SC, Robinson AK, Rodríguez-Quiñones F 2003 Bacterial iron homeostasis. FEMS Microbiol Rev 27:215–237. doi:10.1016/S0168-6445(03)00055-X. 12829269

[59] Rouhier N, Couturier J, Johnson MK, Jacquot JP 2010 Glutaredoxins: roles in iron homeostasis. Trends Biochem Sci 35:43–52. doi:10.1016/j.tibs.2009.08.005. 19811920 PMC2818164

[60] Wei X, Sayavedra-Soto LA, Arp DJ 2004 The transcription of the *cbb* operon in *Nitrosomonas europaea*. Microbiology 150:1869–1879. doi:10.1099/mic.0.26785-0. 15184573

[61] Lorimer GH 1981 The carboxylation and oxygenation of ribulose 1,5-bisphosphate: the primary events in photosynthesis and photorespiration. Annu Rev Plant Physiol 32:349–382. doi:10.1146/annurev.pp.32.060181.002025.

[62] McNevin D, von Caemmerer S, Farquhar G 2006 Determining RuBisCO activation kinetics and other rate and equilibrium constants by simultaneous multiple non-linear regression of a kinetic model. J Exp Bot 57:3883–3900. doi:10.1093/jxb/erl156. 17046981

[63] Jiang D, Khunjar WO, Wett B, Murthy SN, Chandran K 2015 Characterizing the metabolic trade-off in *Nitrosomonas europaea* in response to changes in inorganic carbon supply. Environ Sci Technol 49:2523–2531. doi:10.1021/es5043222. 25546702

[64] Terry KR, Hooper AB 1970 Polyphosphate and orthophosphate content of *Nitrosomonas europaea* as a function of growth. J Bacteriol 103:199–206. 5423370 10.1128/jb.103.1.199-206.1970 PMC248057

[65] Arp DJ, Sayavedra-Soto LA, Hommes NG 2002 Molecular biology and biochemistry of ammonia oxidation by *Nitrosomonas europaea*. Arch Microbiol 178:250–255. doi:10.1007/s00203-002-0452-0. 12209257

[66] Sayavedra-Soto LA, Hommes NG, Russell SA, Arp DJ 1996 Induction of ammonia monooxygenase and hydroxylamine oxidoreductase mRNAs by ammonium in *Nitrosomonas europaea*. Mol Microbiol 20:541–548. doi:10.1046/j.1365-2958.1996.5391062.x. 8736533

[67] Chandran K, Love NG 2008 Physiological state, growth mode, and oxidative stress play a role in Cd (II)-mediated inhibition of *Nitrosomonas europaea* 19718. Appl Environ Microbiol 74:2447–2453. doi:10.1128/AEM.01940-07. 18245236 PMC2293138

[68] Whittaker M, Bergmann D, Arciero D, Hooper AB 2000 Electron transfer during the oxidation of ammonia by the chemolithotrophic bacterium *Nitrosomonas europaea*. Biochim Biophys Acta 1459:346–355. doi:10.1016/S0005-2728(00)00171-7. 11004450

[69] Zorz JK, Kozlowski JA, Stein LY, Strous M, Kleiner M 2018 Comparative proteomics of three species of ammonia-oxidizing bacteria. Front Microbiol 9:938. doi:10.3389/fmicb.2018.00938. 29867847 PMC5960693

[70] Bollmann A, Sedlacek CJ, Norton J, Laanbroek HJ, Suwa Y, Stein LY, Klotz MG, Arp D, Sayavedra-Soto L, Lu M, Bruce D, Detter C, Tapia R, Han J, Woyke T, Lucas SM, Pitluck S, Pennacchio L, Nolan M, Land ML, Huntemann M, Deshpande S, Han C, Chen A, Kyrpides N, Mavromatis K, Markowitz V, Szeto E, Ivanova N, Mikhailova N, Pagani I, Pati A, Peters L, Ovchinnikova G, Goodwin LA 2013 Complete genome sequence of *Nitrosomonas* sp. Is79, an ammonia oxidizing bacterium adapted to low ammonium concentrations. Stand Genomic Sci 7:469–482. doi:10.4056/sigs.3517166. 24019993 PMC3764937

[71] Elmore BO, Bergmann DJ, Klotz MG, Hooper AB 2007 Cytochromes P460 and *c*′-beta; a new family of high-spin cytochromes *c*. FEBS Lett 581:911–916. doi:10.1016/j.febslet.2007.01.068. 17292891

[72] Zahn JA, Arciero DM, Hooper AB, Dispirito AA 1996 Cytochrome *c*′ of *Methylococcus capsulatus* Bath. Eur J Biochem 240:684–691. doi:10.1111/j.1432-1033.1996.0684h.x. 8856071

[73] Liu S, Han P, Hink L, Prosser JI, Wagner M, Brüggemann N 2017 Abiotic conversion of extracellular NH_2_OH contributes to N_2_O emission during ammonia oxidation. Environ Sci Technol 51:13122–13132. doi:10.1021/acs.est.7b02360. 29039187

[74] Zumft WG 1997 Cell biology and molecular basis of denitrification. Microbiol Mol Biol Rev 61:533–616. 9409151 10.1128/mmbr.61.4.533-616.1997 PMC232623

[75] Bueno E, Mesa S, Bedmar EJ, Richardson DJ, Delgado MJ 2012 Bacterial adaptation of respiration from oxic to microoxic and anoxic conditions: redox control. Antioxid Redox Signal 16:819–852. doi:10.1089/ars.2011.4051. 22098259 PMC3283443

[76] Sousa FL, Alves RJ, Pereira-Leal JB, Teixeira M, Pereira MM 2011 A bioinformatics classifier and database for heme-copper oxygen reductases. PLoS One 6:e19117. doi:10.1371/journal.pone.0019117. 21559461 PMC3084760

[77] Hino T, Matsumoto Y, Nagano S, Sugimoto H, Fukumori Y, Murata T, Iwata S, Shiro Y 2010 Structural basis of biological N_2_O generation by bacterial nitric oxide reductase. Science 330:1666–1670. doi:10.1126/science.1195591. 21109633

[78] Sousa FL, Alves RJ, Ribeiro MA, Pereira-Leal JB, Teixeira M, Pereira MM 2012 The superfamily of heme-copper oxygen reductases: types and evolutionary considerations. Biochim Biophys Acta 1817:629–637. doi:10.1016/j.bbabio.2011.09.020. 22001780

[79] Giuffre A, Stubauer G, Sarti P, Brunori M, Zumft WG, Buse G, Soulimane T 1999 The heme-copper oxidases of *Thermus thermophilus* catalyze the reduction of nitric oxide: evolutionary implications. Proc Natl Acad Sci U S A 96:14718–14723. doi:10.1073/pnas.96.26.14718. 10611279 PMC24714

[80] Forte E, Urbani A, Saraste M, Sarti P, Brunori M, Giuffrè A 2001 The cytochrome *cbb3* from *Pseudomonas stutzeri* displays nitric oxide reductase activity. Eur J Biochem 268:6486–6491. doi:10.1046/j.0014-2956.2001.02597.x. 11737203

[81] Pereira MM, Teixeira M 2004 Proton pathways, ligand binding and dynamics of the catalytic site in haem-copper oxygen reductases: a comparison between the three families. Biochim Biophys Acta 1655:340–346. doi:10.1016/j.bbabio.2003.06.003. 15100049

[82] Han H, Hemp J, Pace LA, Ouyang H, Ganesan K, Roh JH, Daldal F, Blanke SR, Gennis RB 2011 Adaptation of aerobic respiration to low O_2_ environments. Proc Natl Acad Sci U S A 108:14109–14114. doi:10.1073/pnas.1018958108. 21844375 PMC3161551

[83] Matsuda Y, Inamori KI, Osaki T, Eguchi A, Watanabe A, Kawabata SI, Iba K, Arata H 2002 Nitric oxide-reductase homologue that contains a copper atom and has cytochrome *c*-oxidase activity from an aerobic phototrophic bacterium *Roseobacter denitrificans*. J Biochem 131:791–800. doi:10.1093/oxfordjournals.jbchem.a003167. 12038974

[84] Zumft WG 2005 Nitric oxide reductases of prokaryotes with emphasis on the respiratory, heme-copper oxidase type. J Inorg Biochem 99:194–215. doi:10.1016/j.jinorgbio.2004.09.024. 15598502

[85] Füssel J, Lücker S, Yilmaz P, Nowka B, van Kessel M, Bourceau P, Hach PF, Littmann S, Berg J, Spieck E, Daims H, Kuypers MMM, Lam P 2017 Adaptability as the key to success for the ubiquitous marine nitrite oxidizer *Nitrococcus*. Sci Adv 3:e1700807. doi:10.1126/sciadv.1700807. 29109973 PMC5665590

[86] Hood-Nowotny R, Umana NHN, Inselbacher E, Oswald-Lachouani P, Wanek W 2010 Alternative methods for measuring inorganic, organic, and total dissolved nitrogen in soil. Soil Sci Soc Am J 74:1018–1027. doi:10.2136/sssaj2009.0389.

[87] Li B, Ruotti V, Stewart RM, Thomson JA, Dewey CN 2010 RNA-Seq gene expression estimation with read mapping uncertainty. Bioinformatics 26:493–500. doi:10.1093/bioinformatics/btp692. 20022975 PMC2820677

[88] Götz F, Pjevac P, Markert S, McNichol J, Becher D, Schweder T, Mussmann M, Sievert SM 2019 Transcriptomic and proteomic insight into the mechanism of cyclooctasulfur- versus thiosulfate-oxidation by the chemolithoautotroph *Sulfurimonas denitrificans*. Environ Microbiol 21:244–258. doi:10.1111/1462-2920.14452. 30362214

