# Supplementary material for: Transcriptomic Response of Nitrosomonas europaea Transitioned from Ammonia- to Oxygen-Limited Steady-State Growth
Source: mSystems. 2020 Jan 14;5(1):e00562-19. doi: 10.1128/mSystems.00562-19 (PMC6967387; doi:10.1128/mSystems.00562-19)
Supplement: TABLE S1 [file mSystems.00562-19-st001.pdf]

Sampling [day]	NH_4^+ [mmol L^{-1}]	NO_2^- [mmol L^{-1}]	N-balance [mmol L^{-1}]	OD600	$gDCW_c$ [gDCW L^{-1}]	NH_3 consumed [mmol L^{-1}]	qNH_3 [mmol $gDCW^{-1}$ h^{-1}]	Y [gDCW mol^{-1}]
0	60	1.49	61.49	0.003	0.00050	0		
1	57.7	1.08	58.78	0.006	0.00099	2.3		
2	55.14	5.68	60.81	0.002	0.00027	4.87		
3	44.05	16.49	60.54	0.038	0.00610	15.95		
4	30.54	29.46	60	0.074	0.01186	29.46		
5	14.32	41.35	55.68	0.118	0.01890	45.68		
6	2.84	59.32	62.16	0.149	0.02381	57.16		
7	2.03	61.22	63.24	0.160	0.02565	57.97	22.60	0.44
8	0.81	60.81	61.62	0.160	0.02560	59.19	23.12	0.43
**9**	**0.68**	**59.32**	**60**	**0.154**	**0.02459**	**59.32**	**24.12**	**0.41**
**10**	**1.35**	**60.41**	**61.76**	**0.146**	**0.02336**	**58.65**	**25.11**	**0.40**
**11**	**0.68**	**57.57**	**58.24**	**0.149**	**0.02376**	**59.32**	**24.97**	**0.40**
12	0.41	60.41	60.81	0.156	0.02488	59.6	23.95	0.42
13	0.81	60.68	61.49	0.146	0.02331	59.19	25.39	0.39
14	0.81	62.7	63.51	0.152	0.02426	59.19	24.40	0.41
15	0.68	59.05	59.73	0.160	0.02560	59.32	23.17	0.43
16	0.54	58.78	59.32	0.158	0.02522	59.46	23.58	0.42
17	7.03	51.08	58.11	0.135	0.02157	52.97		
18	12.84	48.78	61.62	0.114	0.01827	47.16		
19	22.43	41.22	63.65	0.097	0.01554	37.57		
20	30.81	32.43	63.24	0.087	0.01386	29.19		
21	27.43	26.62	54.05	0.089	0.01426	32.57		
22	29.86	24.86	54.73	0.086	0.01374	30.14		
23	30.95	24.59	55.54	0.082	0.01318	29.05	22.03	0.45
24	26.35	24.46	50.81	0.087	0.01392	33.65	24.17	0.41
25	27.7	22.84	50.54	0.081	0.01291	32.3	25.02	0.40
26	28.24	24.19	52.43	0.070	0.01123	31.76	28.28	0.35
27	29.19	24.32	53.51	0.071	0.01130	30.81	27.28	0.37
**28**	**29.73**	**25.54**	**55.27**	**0.070**	**0.01112**	**30.27**	**27.22**	**0.37**
**29**	**28.24**	**24.59**	**52.83**	**0.069**	**0.01096**	**31.76**	**28.98**	**0.35**
**30**	**27.7**	**22.84**	**50.54**	**0.069**	**0.01101**	**32.3**	**29.34**	**0.34**
31	30.14	23.43	53.57	0.070	0.01126	29.86	26.51	0.38
32	30.57	23.86	53.43	0.072	0.01152	29.43	25.55	0.39
