# Supplementary figures and images for: Transcriptomic Response of Nitrosomonas europaea Transitioned from Ammonia- to Oxygen-Limited Steady-State Growth
Source: mSystems. 2020 Jan 14;5(1):e00562-19. doi: 10.1128/mSystems.00562-19 (PMC6967387; doi:10.1128/mSystems.00562-19)

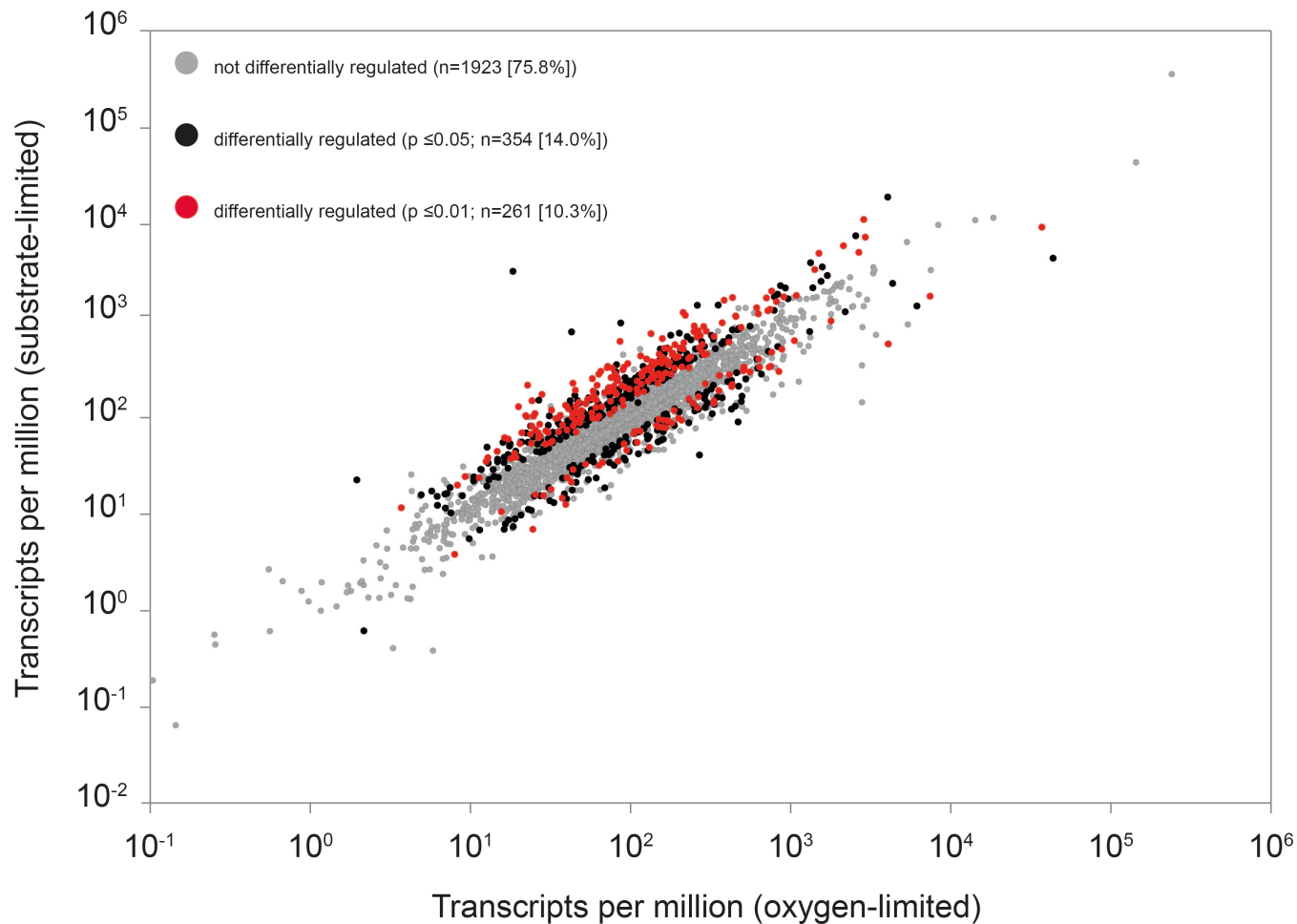

Supplement: FIG S1 [file mSystems.00562-19-sf001.pdf]
