# Supplementary material for: Transcriptomic Response of Nitrosomonas europaea Transitioned from Ammonia- to Oxygen-Limited Steady-State Growth
Source: mSystems. 2020 Jan 14;5(1):e00562-19. doi: 10.1128/mSystems.00562-19 (PMC6967387; doi:10.1128/mSystems.00562-19)
Supplement: TABLE S2 [file mSystems.00562-19-st002.pdf]

Gene ID	Locus tag	Annotation	Fold change	Rank abundance	
				NH_3-limited growth	O_2-limited growth
**Energy conservation**					
amoC	NE1411	Ammonia monooxygenase subunit C singleton	1.9	229	115
amoC	NE0945 / 2064	Ammonia monooxygenase subunit C	-3.3	2	2
amoA	NE0944 / 2063	Ammonia monooxygenase subunit A	-1.6	5	4
amoB	NE0943 / 2062	Ammonia monooxygenase subunit B	-1.3	6	6
amoE	NE0942 / 2061	Ammonia monooxygenase subunit E	1.0	66	81
amoD	NE0941 / NE2060	Ammonia monooxygenase subunit D	-1.2	42	59
haoA	NE0962 / NE2044 / NE2339	Hydroxylamine dehydrogenase subunit A	1.33	245	183
haoB	NE0961 / NE2043 / NE2338	Hydroxylamine dehydrogenase subunit B	1.2	422	387
cycA	NE0960 / NE2042 / NE2337	Cytochrome c-554	-1.07	91	117
cycX	NE0959 / NE2336	Tetraheme cytochrome c-type cm552	-1.03	75	93
ncy	NE0143	Nitrosocyanin	1.7	36	27
cycB	NE0102	Cytochrome c-552	1.3	100	89
cytL	NE0011	Cytochrome P460	-1.6	271	522
cytS	NE0824	Cytochrome c' beta	-2.3**	120	360

Gene ID	Locus tag	Annotation	Fold change	Rank abundance	
				NH_3-limited growth	O_2-limited growth
**Carbon fixation and storage compound metabolism**					
cbbO	NE1918	von Willebrand factor type A / RuBisCO activation protein	-19.71	25	891
cbbQ	NE1919	RuBisCO activation protein	-8.18	26	351
cbbS	NE1920	Ribulose bisphosphate carboxylase small chain	-9.89*	3	16
cbbL	NE1921	Ribulose bisphosphate carboxylase large chain	-4.02**	4	9
cbbR	NE1922	LysR transcription regulator	4.50**	2086	1173
cbbA	NE0324	Fructose-bisphosphate aldolase	1.14	368	348
pykA1	NE0325	Pyruvate kinase	1.18	862	1048
pgk	NE0326	Phosphoglycerate kinase	1.63	207	423
cbbG	NE0327	Glyceraldehyde-3-phosphate dehydrogenase	1.06	266	278
cbbT	NE0328	Transketolase	2.06*	610	304
cah	NE0606	Carbonic anhydrase	-1.20	652	861
cah	NE1926	Carbonic anhydrase	-1.43	310	512
ppk	NE0323	Polyphosphate kinase	2.08**	1084	607
ppx	NE1745	Exopolyphosphatase	-1.05	1085	1223

Gene ID	Locus tag	Annotation	Fold change	Rank abundance	
				NH_3-limited growth	O_2-limited growth
**Universal and oxidative stress response**					
cspD	NE1312	Cold-shock protein	4.9	1172	256
hsp	NE2074	Heat-shock protein	1.6	538	346
sodB	NE0870	Superoxide dismutase	-2.9**	105	421
katA	NE1886	Catalase	-1.5	1414	1756
rubA	NE1426	Rubredoxin	2.78**	965	362
trxA	NE2328	Thioredoxin	1.75**	123	73
**Nitrifier denitrification**					
nirK	NE0924	Nitrite reductase	-4.2**	9	43
ncgC	NE0925	Cytochrome c class I	-2.0	61	156
ncgB	NE0926	Cytochrome c class I	-1.3	57	92
ncgA	NE0927	Multicopper oxidase type I	2.6*	99	30
nsrA	NE0928	Nitrite-sensitive transcriptional repressor	2.1**	656	320
norC	NE2003	Nitric oxide reductase subunit C	1.5*	695	476
norB	NE2004	Nitric oxide reductase subunit B	1.2	749	662
norQ	NE2005	Nitric oxide reductase activation ATPase	1.2	1651	1618
norD	NE2006	Nitric oxide reductase activation protein	1.2	1398	1329

Gene ID	Locus tag	Annotation	Fold change	Rank abundance	
				NH_3-limited growth	O_2-limited growth
**Respiration**					
senC	NE0682	senC (sNOR associated)	2.7**	1356	631
norY	NE0683	sNOR subunit 1	5.5*	428	64
norS	NE0684	sNOR subunit 2	10.8*	1200	102
coxC	NE1013	Cyt c oxidase aa_3 sub 3	1.9**	27	13
coxA	NE1016	Cyt c oxidase aa_3 sub 1	1.7*	50	24
coxB	NE1017	Cyt c oxidase aa_3 sub 2	3.0*	60	16
ctaG	NE1015	Cyt c oxidase aa_3 sub 4	1.9**	86	45
petA	NE0809	Ubiquinol-cytochrome c reductase	3.0**	389	110
petB	NE0810	Cytochrome b	2.2*	112	44
petC	NE0811	Cytochrome c	1.1	114	118
nuoN	NE1764	NADH-quinone oxidoreductase subunit N	1.2	494	448
nuoM	NE1765	NADH:ubiquinone oxidoreductase subunit M	1.2	659	612
nuoL	NE1766	NADH:ubiquinone oxidoreductase subunit L	1.3	391	323
nuoK	NE1767	NADH-quinone oxidoreductase subunit K	-1.2	498	681
nuoJ	NE1768	NADH:ubiquinone oxidoreductase subunit J	-1.0	336	381

Gene ID	Locus tag	Annotation	Fold change	Rank abundance	
				NH_3-limited growth	O_2-limited growth
nuoI	NE1769	NADH-quinone oxidoreductase subunit I	-2.8	131	500
nuoH	NE1770	NADH-quinone oxidoreductase subunit H	-1.6**	119	236
nuoG	NE1771	NADH dehydrogenase subunit G	-1.1	240	288
nuoF	NE1772	NADH dehydrogenase	1.0	355	379
nuoE	NE1773	NADH dehydrogenase	1.4**	485	377
nuoD	NE1774	NADH-quinone oxidoreductase subunit D	1.2*	321	299
nuoC	NE1775	NADH-quinone oxidoreductase subunit C	-1.4	253	424
nuoB	NE1776	NADH-quinone oxidoreductase subunit B	1.0	323	349
nuoA	NE1777	NADH:ubiquinone oxidoreductase subunit A	1.2	201	173
atpI	NE0199	ATP synthase unit I	5.2**	516	85
atpB	NE0200	ATP synthase subunit A	2.8*	345	148
atpE	NE0201	ATP synthase subunit C	1.1	87	96
atpF	NE0202	ATP synthase subunit B	-1.2	244	342
atpH	NE0203	ATP synthase subunit delta	1.0	208	221
atpA	NE0204	ATP synthase subunit alpha	-1.1	133	158
atpG	NE0205	ATP synthase subunit gamma	1.2	390	343
atpD	NE0206	ATP synthase subunit beta	1.1	102	103
atpC	NE0207	ATP synthase epsilon chain	1.3	101	87
